# Fuzzy Modeling to Predict Severely Depressed Left Ventricular Ejection Fraction following Admission to the Intensive Care Unit Using Clinical Physiology

**DOI:** 10.1155/2015/212703

**Published:** 2015-08-05

**Authors:** Rúben Duarte M. A. Pereira, Cátia M. Salgado, Andre Dejam, Shane R. Reti, Susana M. Vieira, João M. C. Sousa, Leo A. Celi, Stan N. Finkelstein

**Affiliations:** ^1^IDMEC, Instituto Superior Técnico, Universidade de Lisboa, 1049-001 Lisboa, Portugal; ^2^Engineering Systems Division, Massachusetts Institute of Technology, Cambridge, MA 02139, USA; ^3^Beth Israel Deaconess Medical Center, Cardiology Division and Harvard Medical School, Boston, MA 02215, USA; ^4^Beth Israel Deaconess Medical Center, Division of Clinical Informatics and Harvard Medical School, Boston, MA 02215, USA; ^5^Beth Israel Deaconess Medical Center, Pulmonary Division and Harvard Medical School, Boston, MA 02215, USA; ^6^Massachusetts Institute of Technology, Harvard-MIT Division of Health Sciences & Technology, Cambridge, MA 02139, USA

## Abstract

Left ventricular ejection fraction (LVEF) constitutes an important physiological parameter for the assessment of cardiac function, particularly in the settings of coronary artery disease and heart failure. This study explores the use of routinely and easily acquired variables in the intensive care unit (ICU) to predict severely depressed LVEF following ICU admission. A retrospective study was conducted. We extracted clinical physiological variables derived from ICU monitoring and available within the MIMIC II database and developed a fuzzy model using sequential feature selection and compared it with the conventional logistic regression (LR) model. Maximum predictive performance was observed using easily acquired ICU variables within 6 hours after admission and satisfactory predictive performance was achieved using variables acquired as early as one hour after admission. The fuzzy model is able to predict LVEF ≤ 25% with an AUC of 0.71 ± 0.07, outperforming the LR model, with an AUC of 0.67 ± 0.07. To the best of the authors' knowledge, this is the first study predicting severely impaired LVEF using multivariate analysis of routinely collected data in the ICU. We recommend inclusion of these findings into triaged management plans that balance urgency with resources and clinical status, particularly for reducing the time of echocardiographic examination.

## 1. Introduction

The measurement of LVEF is a well-established clinical parameter that has essential diagnostic, therapeutic, and prognostic implications, particularly in the settings of coronary artery disease and heart failure [1–3]. Several studies have addressed clinical outcomes relating to LVEF measurements [4–10]. These have demonstrated a general LVEF threshold level of 45%, below which there is a generally linear relationship between LVEF decrease and increase in mortality and cardiac events, although recent studies have indicated that a preserved ejection fraction does not necessarily mean freedom from risk [6, 11–14].

Patients with LVEF ≤ 25% constitute a particularly sick heart failure population [6] and extreme caution has to be exercised in treating these patients [4, 5, 7–10]. Cardiac dysfunction as evidenced by reduced ejection fraction is present in most patients with severe sepsis and septic shock [15]. Fluid resuscitation therapy may be lifesaving in these cases, especially in the early phases of treatment. However, patients with a very low ejection fraction may be asymptomatic due to processes such as remodeling, which can precede the development of symptoms [12]. Early detection to guide fluid resuscitation in the intensive care unit (ICU) is crucial.

Noninvasive technologies such as echocardiography, computed tomography, magnetic resonance imaging, gated single photon emission computed tomography, and multigated acquisition scanning are used to assess LVEF [16]. Each of these techniques is resource intensive requiring special equipment and specialized technicians, which might delay the procedure in situations such as examining at night or at the weekend. They also have varying degrees of patient invasiveness and consequent risk of complications, progressing from the least invasive echocardiography to the most invasive catheter placement [17]. Transthoracic echocardiography is the most feasible, safest, and cheapest method to assess LVEF and has been used routinely for this purpose for decades [16]. The popularity of this technique and its attendant costs have led to growing concern about potential overuse of this important technology. Studies on the trends in echocardiography utilization in the Veterans Administration Healthcare System show that while utilization on a per-patient basis remained relatively stable, increasing only by 2.7% between 2000 and 2007, the actual number of echocardiograms between 2000 and 2007 increased by 112.2% [18]. Limited transthoracic echocardiogram (LTTE) represents an attractive alternative to the typical transthoracic echocardiogram (TTE) since it can be performed with minimal training and significantly reduces the examination time from the usual 45–60 minutes to less than 5 minutes [19].

The aim of this study is to use variables easily acquired in the intensive care unit (ICU) to develop a predictive model for assessing severely depressed LVEF as early as possible following ICU admission. We envision that the integration of the predictive model in a healthcare's decision support system will allow typical TTE to be substituted by LTTE for confirmation of the predicted results.

## 2. Methods

This retrospective study made use of the MIMIC II (Multiparameter Intelligent Monitoring in Intensive Care) database [20]. MIMIC II contains deidentified patients' data [21] and is publicly available on the* PhysioNet* website (http://www.physionet.org/). It encompasses a diverse and very large population of ICU patients from the Beth Israel Deaconess Medical Center, in Boston, dating from 2001. The temporal resolution of the data, including administrative data, laboratory results, clinical notes, bedside monitor trends, and waveforms, allows a diverse range of analytic studies spanning epidemiology, clinical decision analysis, and decision support development [20]. Newer versions of the database are released as more patient records are archived. The downloadable files of MIMIC II Clinical Database version 2.5 used in this work contain data for 26,655 subjects.

### 2.1. Data Extraction

We extracted a total of 17 features, which are presented in the list below, including demographics and physiological variables, using a standard variable selection process that seeks to maximize the amount of data within any given variable versus maintaining robust numbers and statistical power (the rule of thumb is that the number of patients should be about 10 times higher than the number of variables) [22]. Opinion of physicians and existing literature mandated the inclusion of heart rate and blood pressure, acknowledging the tight relation with cardiac function monitoring. Including the next most acquired variable would result in a significant drop in the amount of patients with data available for all variables considered.

*List of Demographic Information and Physiological Variables Extracted from the Database*

 Heart rate (beats per minute).
 Diastolic noninvasive blood pressure: diastolic NBP (mmHg).
 Systolic noninvasive blood pressure: systolic NBP (mmHg).
 Respiratory rate (breaths per minute).
 Oxygen saturation in the blood (%).
 Temperature (°C).
 Blood urea nitrogen: BUN (mg/dL).
 Carbon dioxide concentration in blood (mmol/L).
 Glucose (mg/dL).
 Hemoglobin (mmol/L).
 Platelets (×10^9^ cells/L).
 Potassium (mmol/L).
 Sodium (mmol/L).
 White blood cells (×10^3^ cells/*μ*L).
 Age (years).
 Weight on admission (kg).
 Gender.

**Figure 1:**
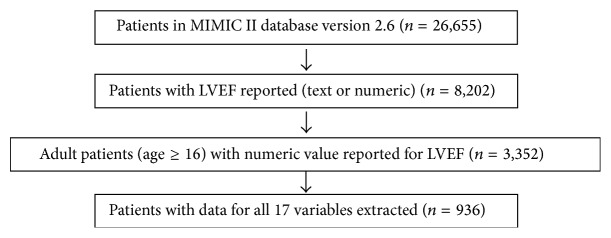
Flow chart of inclusion criteria and number of patients used to define the cohort. LVEF indicates left ventricular ejection fraction; ICU indicates intensive care unit.

In MIMIC II, LVEF assessments are reported in clinical notes, either in textual (e.g., “normal LVEF”) or numeric forms. Text mining techniques were used to retrieve the numeric values such that whenever a numeric interval was reported, the mean value was computed. The values were validated by the authors for abnormal occurrences, regarding misspelling and values that fell outside the possible physiologic range. Inclusion criteria included adult patients reporting a numeric LVEF value and data for each of the 17 features (Figure 1). Textual information was not considered for this study.

### 2.2. Preprocessing

A summary of the data processing steps is as follows: (1) LVEF was separated into two classes using 25% as the reference value [6]; (2) outliers were addressed using expert knowledge; (3) mean values were computed to obtain input data; (4) data were normalized using the min-max procedure.

**Figure 2:**
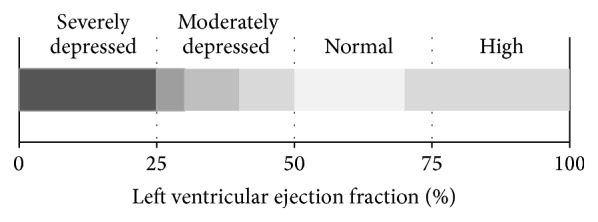
Domains of left ventricular ejection fraction associated with clinical risk.

Figure 2 illustrates the several domains of LVEF described in the literature [23–25].

The approach followed in this study consists of a dichotomous classification problem. Patients with LVEF ≤ 25% were regarded as the “severely depressed LVEF” class and the remaining as the “non-severely depressed LVEF” class. Several groups have shown consistency in observations using a 25% LVEF cut-off value for defining severely depressed groups [4, 5, 7–10]. Using this criterion, we included 115 patients in the positive class (12.3% of the extracted dataset).

After retrieving the cohort, data was filtered for outliers. Methods that process the data based only on its statistical properties (e.g., quartile distribution, standard deviation, or confidence intervals) aim to determine outliers with no prior knowledge of the data [26]. In this case, filtering was made using medical knowledge, and measurements that exceeded possible physiological boundaries for each variable were eliminated. The fuzzy modeling strategy used subsequently is able to weight each nondeleted observation according to the overall distribution [27]; that is, the membership functions inherent to the modeling strategy reflect whether an observation is frequent or not, decreasing the impact of preprocessing outliers.

Data normalization consisted in the attribution of the value 0 to the minimum value observed and the value 1 to the maximum value observed. All values were then rescaled accordingly.

For purposes of determining whether two groups are significantly different from each other, we assumed the data follows normal distributions. The family distribution of each input variable was assessed through the Pearson System to confirm our assumption [28]. Two-sample *t*-test was used to test the null hypothesis that data in the two groups are independent random samples from normal distributions with equal means but unknown variances, against the alternative that the means are not equal.

### 2.3. Modeling

Fuzzy modeling is a tool that allows an approximation of nonlinear systems when there is little or no previous knowledge of the system to be modeled, providing linguistic interpretation in the form of rules. This interpretation is particularly appealing in clinical related scenarios as it allows unraveling the most striking rules in order to seek expert understanding [29–31]. In this work, we used first order Takagi-Sugeno (TS) fuzzy models [14] to perform classification. A TS fuzzy model is a fuzzy rule-based model where the rule consequents are functions of the model input. Each rule *k* has a different function yielding a different value *y* ^*k*^ for the output. The simplest consequent function is the linear affine form:

(1) $$\begin{array}{c} R^{k}:\text{If }x\text{ is }A^{k}\text{ then }y^{k}={\left(\;a^{k}\;\right)}^{T}x+b^{k}, \end{array}$$

where *R* ^*k*^ denotes the *k*th rule, **x** the vector of antecedent variables, *A* ^*k*^ the (multidimensional) antecedent fuzzy sets, *y* ^*k*^ the one-dimensional consequent variable of the *k*th rule, **a** ^*k*^ a vector of parameters, and *b* ^*k*^ a scalar offset that relates the antecedent fuzzy sets with the consequents.

Fuzzy clustering allows one piece of data to belong to two or more clusters with different degrees. The approach used in this paper builds a TS inference model based on the Gustafson-Kessel fuzzy clustering algorithm [37]. This algorithm extends the traditional fuzzy c-means (FCM) [38] by introducing an augmented form of the Euclidean distance that allows the detection of clusters of different geometrical shapes and orientation. Each cluster is used to create one rule and upon evaluation of the model all rules created are activated, each according to the membership degree of the sample to the cluster. A continuous real output is returned based on the weighted sum of the outputs of each rule:

(2) $$\begin{array}{c} Output=\frac{\sum_{i=1}^{R}\beta_{i}y_{i}}{\sum_{i=1}^{R}\beta_{i}}, \end{array}$$

where *β*_*i*_ is the degree of activation of each rule and *R* is the number of rules.

Logistic regression (LR) is a conventional statistical method, within the medical field, to model the probability of binary events (e.g., treatment response). It computes a linear classifier to predict the probability, (*Y* = 1∣**x**) = *π*(**x**), of an event that depends on *p* independent variables, using a logit function [37]. The logistic regression model is given by

(3) $$\begin{array}{c} \pi \left(\;x\;\right)=\frac{e^{\beta_{0}+\beta_{1}x_{1}+\beta_{2}x_{2}+\cdots +\beta_{p}x_{p}}}{1+e^{\beta_{0}+\beta_{1}x_{1}+\beta_{2}x_{2}+\cdots +\beta_{p}x_{p}}}, \end{array}$$

where **x** = *x*_1_, *x*_2_,…, *x*_*p*_ represents the whole set of variables and *β* = *β*_0_, *β*_1_,…, *β*_*p*_ are the regression coefficients. The logit function is defined as follows:

(4) $$\begin{array}{c} g\left(\;x\;\right)=\beta_{0}+\beta_{1}x_{1}+\beta_{2}x_{2}+\cdots +\beta_{p}x_{p}. \end{array}$$

Dichotomous classification is obtained by applying a threshold to the output of each model. This threshold balances accuracy (correct classification rate), sensitivity (true positive classification rate), and specificity (true negative classification rate). The performance of the models was evaluated in terms of area under the receiver operating characteristic curve (AUC), accuracy, sensitivity, and specificity.

### 2.4. Study Design

The dataset was initially divided into two subsets of the same size: one for feature selection (FS) and the other for model assessment (MA). In the FS subset, a combination of feature selection with fuzzy modeling or logistic regression was performed to find the subset of features that produces the best AUC. Models for performing feature selection were iteratively built and evaluated using 5-fold cross validation, repeated for 500 random configurations of the folds. The set of features more often selected in the 500 repetitions was chosen as the best set. The validity and robustness of the model was also assessed using 5-fold cross validation, repeated for 50 random configurations of the folds, in the MA dataset, using the best set of features identified. Results were averaged over the rounds.

The whole datasets corresponding to each time interval in the ICU were used to perform exploratory tests, using 5-fold cross validation repeated for 2 random configurations of the folds. Results showed that using a number of clusters between 2 and 10 does not change the performance of the models. Thus, in order to facilitate the clustering of patients and consequent interpretation of the models, 2 clusters were used.

### 2.5. Assessment of the Best Predicting Interval following ICU Admission

In order to assess the most suitable time interval following ICU admission to predict severely depressed LVEF, we used an exploratory set to develop fuzzy classification models. This set comprises the 5 physiological variables most regularly acquired within the ICU stay and 2 demographic constants consistently acquired in each admission to the ICU. These variables are heart rate, noninvasive diastolic and systolic blood pressure, respiratory rate, and oxygen saturation, coupled with age and weight. This approach aims to obtain equal amount of samples in all variables across all time intervals considered for comparison, since some of the variables are only acquired once a day.

We compared the AUC of different, nonoverlapping, 6-hour intervals during the first 30 hours of admission. The number and type of variables increased as the time intervals increased up to 6 hours, starting at ICU admission, and remained stable thereafter. Therefore, we used as input for the models the mean value of each 6-hour measurement in each variable. Modeling based on observations during the first hour of admission was also performed at this stage.

### 2.6. Sequential Forward Selection

To fine-tune performance, we aimed to evaluate different combinations of variables from the preprocessed dataset containing data for all extracted variables. Using the data contained in the best interval, we explored the use of all the extracted physiological variables. The sequential forward selection (SFS) method with criteria based on the AUC was used. The SFS method sequentially adds features to the best set previously evaluated until a stopping criterion is achieved (e.g., no improvement in performance).

### 2.7. Interpretation of Rules

After selection and validation of the best set of variables, we built an inference model based on the observations within the best time interval in the ICU. By assessing the parameters from the inference model, along with the membership functions depicted, it is possible to extract the numeric expressions (rules) that are computed in order to obtain the classification.

The higher the amount of patients from one class that presents higher membership degree to only one of the clusters, the higher the certainty that a patient belonging to that cluster belongs to that class. Therefore, we can conclude that if the membership degree is higher in all the input variables used to develop the model, then the class most possibly observed will be the class predominant in that cluster.

## 3. Results

Table 1 represents the baseline characteristics of the final set of patients regarding the considered classes, including the Simplified Acute Physiology Score (SAPS) and the Sequential Organ Failure Assessment (SOFA) score.

**Figure 3:**
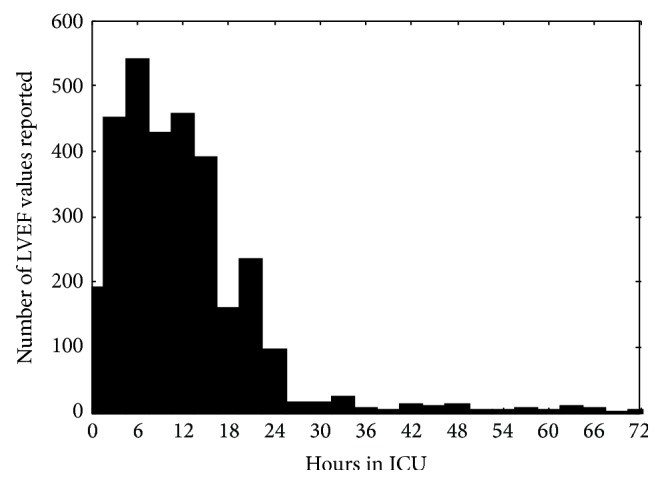
Distribution of the valid numerical values reported for LVEF during the first 3 days of stay in the ICU, referring to the time of ICU admission (0 hours).

Figure 3 presents the distribution of the number of LVEF values reported within the first 3 days of ICU stay. The data shows that 90% of the LVEF assessments are performed within 24 hours after ICU admission and 95% are performed within the ICU stay.

### 3.1. Assessment of the Best Predicting Interval following ICU Admission Using Fuzzy Modeling

**Figure 4:**
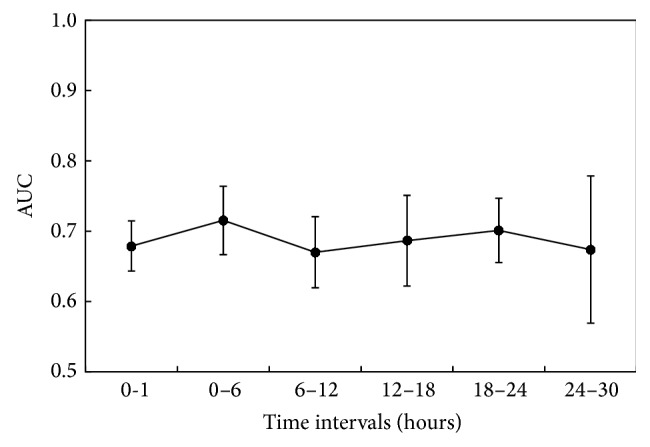
Variation of AUC using different, nonoverlapping, 6-hour intervals during the first 30 hours after ICU admission.

Comparing different, nonoverlapping, 6-hour intervals during the first 30 hours of admission (Figure 4 and Table 2), we observe a decrease in AUC from the first 6-hour interval to the 6–12 hours' post-ICU admission interval (*P* value = 0.089). The AUC in the 0–6 hours' interval is 0.72 ± 0.05, decreasing to 0.67 ± 0.05 in the 6–12 hours' interval. Though not significant, this is most likely due to initiation of therapy. The performances of the time intervals 12–18 and 18–24 hours are still inferior to that of the first 6 hours after ICU admission (AUC are 0.69 ± 0.06 and 0.70 ± 0.05 and *P* values of the two-sample *t*-test are 0.105 and 0.599, resp.). The *P* value of the two-sample *t*-test comparing the 24–30 hours' period and the first 6 hours of admission is 0.339; that is, the performance of the model past 24 hours in the ICU is not statistically different. The AUC 24 hours after ICU admission is 0.67 ± 0.10. The AUC obtained using only 1 hour of measurements is 0.68 ± 0.04, which is lower than that of the first 6 hours after ICU admission (*P* value = 0.195).

We can conclude that the time frame comprising the first 6 hours following ICU admission is the best both to predict severely depressed LVEF and to provide the earliest assessment of LVEF in the ICU. This interval was therefore used in the next steps to fine-tune the model.

### 3.2. Sequential Forward Selection

The set selected by SFS in combination with fuzzy modeling is composed of seven variables: systolic noninvasive blood pressure, *x*_1_, respiratory rate, *x*_2_, blood urea nitrogen levels, *x*_3_, hemoglobin, *x*_4_, sodium, *x*_5_, white blood cells, *x*_6_, and weight on admission, *x*_7_. The set selected by SFS combined with LR is composed of six variables: noninvasive blood pressure (diastolic and systolic), respiratory rate, blood urea nitrogen levels, potassium, and age on admission.

The performance results are shown in Table 3. From this table, it follows that, for the FS subset, the best combination of variables was obtained through the combination of SFS with fuzzy modeling, which resulted in an AUC of 0.71 ± 0.07. The traditional LR method performed only moderately in identifying patients with severely depressed LVEF with an AUC of 0.67 ± 0.07. Models are statistically different (*P* < 0.001).

### 3.3. Interpretation of Rules

**Figure 5:**
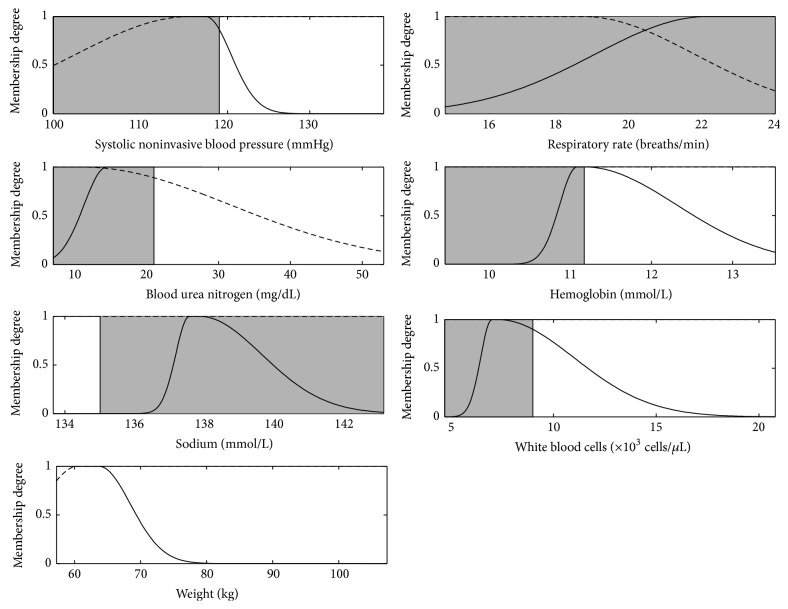
Membership functions obtained for assessing severely depressed LVEF. Lower and upper bounds accepted for normal values ranges are also depicted (grey zone). The solid-lined cluster corresponds to the antecedents of Rule 1 (*A* ^*i*,1^) and the dash-lined cluster corresponds to those of Rule 2 (*A* ^*i*,2^).

The membership functions obtained for each variable are depicted in Figure 5. The membership functions depicted by the continuous line correspond to Rule 1. The dash-lined membership functions correspond to Rule 2. The normal physiological range values for each physiological variable are depicted in Table 4 to support further linguistic interpretation of the rules.

The model created consists of nonlinear combinations and therefore we cannot investigate any of the variables separately from the others. We were able to extract two distinct rules that can constitute a first assessment of severely depressed LVEF in the ICU. The rules are as follows.

**Rule 1 .**

If *x*_1_ is *A* ^1,1^ and *x*_2_ is *A* ^2,1^ and *x*_3_ is *A* ^3,1^ and *x*_4_ is *A* ^4,1^ and *x*_5_ is *A* ^5,1^ and *x*_6_ is *A* ^6,1^ and *x*_7_ is *A* ^7,1^, then

(5) y1=−0.0093·x1+0.0670·x2+0.2700·x3+0.5000·x4−1.200·x5+0.4000·x6+0.0540·x7+0.5000.

**Rule 2 .**

If *x*_1_ is *A* ^1,2^ and *x*_2_ is *A* ^2,2^ and *x*_3_ is *A* ^3,2^ and *x*_4_ is *A* ^4,2^ and *x*_5_ is *A* ^5,2^ and *x*_6_ is *A* ^6,2^ and *x*_7_ is *A* ^7,1^, then

(6) y2=−0.4300·x1+0.3100·x2+0.5000·x3+0.2400·x4−0.0280·x5−0.1500·x6−0.3600·x7+0.1400.

The output is computed by calculating the weighted* sum* of the results of the activation of both rules and the class is obtained by applying a cut-off value to the output. Class** 1** corresponds to “severely depressed LVEF” and class** 0** to “non-severely depressed LVEF.”

## 4. Discussion

Several works have addressed the characteristics of populations with severely depressed LVEF versus other LVEF values regarding comorbidities and past medical interventions [8–10]. However, to the best of our knowledge, this is the first study predicting severely impaired LVEF using routinely collected data in the ICU. Individual characteristics identified in this work (Table 1) agree with past publications [6, 11, 13]. Patients in the severely impaired LVEF group tend to be males and to present higher heart rate, lower systolic blood pressure, and higher blood urea nitrogen levels.

Variables obtained during the first 6 hours of ICU admission can best predict the presence of severely depressed LVEF. Additionally, satisfactory predictive performance can be achieved using variables obtained within the first hour post-ICU admission and depending on the urgency of treatment, physicians might act upon this time interval in critical situations. The developed fuzzy model constitutes a preliminary assessment to perform a limited protocol examination.

The first hours are crucial for treatment outcome, particularly regarding assessment of tissue perfusion in order to direct fluid resuscitation therapy. In patients with sepsis, for example, early intervention with aggressive fluid resuscitation has repeatedly been associated with improved outcome [39]. Knowledge of an ejection fraction and susceptibility to respiratory failure may improve patient outcomes by guiding fluid management. For this fact, it is expected that within a short time frame following ICU admission most of the data necessary for accurate prediction of poor LVEF is already acquired, as observed in the study.

One of the benefits of using fuzzy logic is the creation or rules that can then be informative for potential clinical guidelines. However, the certainty around these rules can only be assumed as far as the data used allows. Generalization of the models must take into account each of the issues addressed in the steps conducted in this study, such as characteristics of the dataset, or the established protocols in the ICU that influence the amount and frequency of data acquired.

## 5. Conclusions

This work is focused on building a predictive model for application in the ICU for rapid identification of patients with severely depressed LVEF. It is expected that the use of readily available clinical parameters for an early assessment of severely depressed LVEF will improve the outcomes associated with the administration and management of fluids in the ICU. The fuzzy model implemented in this work is able to predict an LVEF ≤ 25% with an AUC of 0.71 ± 0.07, outperforming the traditional logistic regression model (AUC 0.67 ± 0.07).

Further efforts must be made to validate the results in other databases and in real clinical scenarios. This would include prospective work to determine how accurate the predictions are in different specific environments and impact studies to see whether such prediction will influence clinician decision-making and/or improve hospital and treatments cost efficiency.

Also, it would be useful to investigate the performance of predictive models using baseline variables collected at the time of ICU admission.

## Figures and Tables

**Table 1:** Baseline demographic and clinical characteristics within the first 6 hours of ICU admission in the cohort regarding patients with LVEF ≤ 25% and LVEF > 25%.

Variables	LVEF ≤ 25%	LVEF > 25%	*P* values
	115 patients	821 patients	
Braden score	14.4 ± 2.6	14.8 ± 2.6	0.121
Total Glasgow coma score	12.1 ± 3.9	12.5 ± 3.6	0.345
Heart rate (beats/minute)	88.6 ± 17.8	84.2 ± 19.2	0.021
Diastolic NBP (mmHg)	59.2 ± 12.0	60.8 ± 13.6	0.249
Systolic NBP (mmHg)	110.2 ± 16.9	120.1 ± 20.2	<0.001
Respiratory rate (breaths/minute)	21.0 ± 4.8	19.2 ± 4.6	<0.001
Oxygen saturation (%)	96.8 ± 3.9	97.2 ± 2.9	0.278
Temperature (°C)	36.6 ± 0.8	36.7 ± 0.8	0.474
Blood urea nitrogen (mg/dL)	37.6 ± 26.4	28.9 ± 22.3	<0.001
Carbon dioxide (mmol/L)	22.9 ± 5.0	23.4 ± 5.0	0.266
Glucose (mg/dL)	172.2 ± 78.1	151.9 ± 65.7	0.003
Hemoglobin (mmol/L)	11.9 ± 2.4	11.4 ± 2.0	0.032
Platelets (×10^3^ cells/*μ*L)	244.2 ± 112.5	235.2 ± 108.3	0.408
Potassium (mmol/L)	4.3 ± 0.6	4.2 ± 0.7	0.050
Sodium (mmol/L)	137.8 ± 5.2	138.5 ± 4.7	0.139
White blood cells (×10^3^ cells/*μ*L)	12.7 ± 5.6	12.8 ± 8.3	0.971
Age (years)	70.6 ± 15.1	67.3 ± 16.4	0.045
Weight on admission (kg)	77.1 ± 18.6	83.0 ± 25.7	0.018
SAPS I score on admission	13.9 ± 5.9	13.4 ± 5.5	0.428
SOFA score on admission	6.3 ± 4.2	5.4 ± 4.3	0.036
Males (%)	74 (64.4)	432 (52.6)	0.018

Data are presented as mean ± standard deviation, except for males where data is presented as number of patients (% of the total dataset).

**Table 2:** Performance of the fuzzy model using different, nonoverlapping, 6-hour intervals during the first 30 hours after ICU admission.

	1st hour	0–6 hours	6–12 hours	12–18 hours	18–24 hours	24–30 hours
AUC	0.68 ± 0.04	0.72 ± 0.05	0.67 ± 0.05	0.69 ± 0.06	0.70 ± 0.05	0.67 ± 0.10
Accuracy	0.64 ± 0.02	0.65 ± 0.03	0.63 ± 0.04	0.64 ± 0.06	0.66 ± 0.03	0.65 ± 0.04
Sensitivity	0.65 ± 0.03	0.65 ± 0.04	0.64 ± 0.04	0.64 ± 0.07	0.66 ± 0.04	0.65 ± 0.04
Specificity	0.57 ± 0.08	0.64 ± 0.12	0.59 ± 0.05	0.64 ± 0.08	0.66 ± 0.14	0.60 ± 0.19

**Table 3:** Performance of the fuzzy and LR models within the first 6 hours after ICU admission using the best set of variables selected through sequential forward selection.

	Fuzzy model Best SFS set	Logistic regression model Best SFS set
AUC	0.71 ± 0.07	0.67 ± 0.07
Accuracy	0.68 ± 0.05	0.66 ± 0.05
Sensitivity	0.63 ± 0.14	0.62 ± 0.14
Specificity	0.68 ± 0.06	0.66 ± 0.06

**Table 4:** Normal range of values for each physiological variable.

Variables	Normal range
Systolic NBP (mmHg)	90 to 119 [26]
Respiratory rate (breaths/minute)	12 to 24 [32]
Blood urea nitrogen (mg/dL)	7 to 21 [33]
Hemoglobin (mmol/L)	8.56 to 11.17 (7.51 to 9.37 for women) [34]
White blood cells (×10^3^ cells/*μ*L)	3.5 to 9 [35]
Sodium (mmol/L)	135 and 145 [36]
